# Optimization of Key Factors in Serum Free Medium for Production of Human Recombinant GM-CSF Using Response Surface Methodology 

**DOI:** 10.22037/ijpr.2020.112322.13681

**Published:** 2019

**Authors:** Nazanin Ghasemi, Mojgan Bandehpour, Javad Ranjbari

**Affiliations:** a *Department of Immunology, School of Medicine, Shahid Beheshti University of Medical Sciences, Tehran, Iran. *; b *Department of Medical Biotechnology, School of Advanced Technologies in Medicine, Shahid Beheshti University of Medical Sciences, Tehran, Iran.*

**Keywords:** Serum free medium, RSM, Optimization, GM-CSF, Key factors

## Abstract

Researchers add serum to a classical medium at concentrations of 5 to 10% (v/v) to grow cells *in-vitro *culture media. Unfortunately, serum is a poorly defined culture medium component as its composition can vary considerably while serum-free cell culture media are an excellent alternative to standard serum-containing media and offer several major advantages. Advantages of using serum-free media include a lower risk of infectious agents, lower risk of interfering components, less contaminant, avoids ethical issues. According to previous studies insulin, selenium, transferrin and glucose are important component of serum that affect cell growth. In the present study, we optimized amount of these factors in order to serum free culture medium fabrication. Response surface methodology (RSM) was employed for optimization of key factors in serum free medium to enhance recombinant human GM-CSF (rhGM-CSF) production in CHO cell line. Four important process parameters including insulin concentration (0-2 g/L), transferrin concentration (0-1 g/L), selenium concentration (0-0.001 g/L) and glucose concentration (0-5 g/L) were optimized to obtain the best response of rhGM-CSF production using the statistical Box–Behnken design. The experimental data obtained were analyzed by analysis of variance (ANOVA) and fitted to a second-order polynomial equation using multiple regression analysis. Numerical optimization applying desirability function was used to identify the optimum conditions for maximum production of rhGM-CSF. The optimum conditions were found to be insulin concentration = 1.1 g/L, transferrin concentration = 0.545 g/L, selenium concentration = 0.000724 g/L and glucose = 1. 4 g/L. Maximum rhGM-CSF production was found to be 3.5 g/L.

## Introduction

Glycosylated rhGMCSF is an 18-32 kDa protein depends on glycosylation degree that was initially characterized as a factor that can support the *in-vitro *colony formation of granulocytemacrophage progenitors. It is also a growth factor for erythroid, megakaryocyte, and eosinophil progenitors. As the biotechnology industry has rapidly expanded in recent years, the expression of a spectrum of recombinant proteins in different systems for a wide variety of purposes has been a major feature and challenge. The majority of therapeutic proteins have been produced in either mammalian cell-culture systems, with Chinese hamster ovary (CHO) cells representing the most common system, or in *Escherichia coli*. A variety of alternative expression systems are also being developed and evaluated (1). It seems to be a significant increase in the application of mammalian cells for proteins production. Expression systems utilizing mammalian cells for recombinant proteins are able to introduce proper protein folding, post-translational modifications, and product assembly, which are important for complete biological activity. The production of proteins in appropriate quantity and quality is an essential requirement of the present time. Enhanced production and, consequently, lower costs of the final product is commercially important (2, 3). Various factors affect the yield and quality of recombinant proteins in mammalian systems. The most important factors in this regard include expression system type, structural changes in the recombinant gene can result in loss of expression, purification process and posttranslational modifications like glycosylation can affect the folding, aggregation, and solubility of proteins (4). Composition of culture media is one of the most important factors affect the cell growth and, consequently, recombinant protein yield and quality (5-8). In industrial applications culture media must support high viable cell densities while also increase product quality and decrease production costs. For decades, researchers use complex culture media for the cultivation of animal cells were typically composed of plasma, serum, or tissue extracts. Serum is the liquid fraction of clotted blood, depleted of cells, fibrin and clotting factors, but containing a large number of nutritional and macromolecular factors like proteins. For example, albumin is the major component of the fetal bovine serum. Fetal bovine serum also contains small molecules like amino acids, sugars, lipids, and hormones (9, 10). The complex, undefined nature of these supporting media resulted in variability, increased risk of contamination, and prevented the clarification of the specific nutrient components required to support the growth of cells in the medium. For these reasons, many research groups have developed serum-free, chemically defined cell culture media through analysis of cell nutrient demands and serum composition, and identification of the key ingredients needed to support growth (11). In serum-free media we have a chemically defined medium with controlled conditions *in-vitro*. Also, reduced variability in qualitative and quantitative culture medium composition and removal of a potential source of microbial contamination has been reported. Serum free media reduce interferer proteins and facilitate isolation and purification of target recombinant protein. Serum composition analysis shows the insulin, selenium and transferrin are the most important of serum ingredients that enhance cell proliferation and decrease the serum requirement of many cell types (12). Multivariate statistic techniques are used to improve the performance of a system like serum free medium to obtain the best composition of its elements and following that the best cell growth and protein production. One of the most relevant multivariate statistic techniques for optimization of analytical procedures is Response Surface Methodology (RSM). RSM is a collection of mathematical and statistical tools for creating a model to obtaining maximum information from a minimal number of experiments by varying simultaneously all the process key factors (12, 13). 

According to above reasons and last studies we decided to design a serum free medium by optimize amount of insulin, selenium, transferrin and glucose by RSM methodology and select the best composition of these factor for maximum production of rhGM-CSF.

## Experimental

*Materials*

CHO-K1 cell line was purchased from American Type Culture Collection. Dulbecco’s Modified Eagle Medium/Ham’s F12 (DMEM/Ham’s F12), fetal Bovine serum (FBS) and penicillin/streptomycin were purchased from Gibco, USA. pcDNA3. lipofectamine, hygromycin, trypsin, EDTA, Tris, SDS, coomassie blue G250, glacial acetic acid, glycerol, β-mercaptoethanol, bromphenol blue and protein molecular weight marker were ordered from Sigma, USA. rhGM-CSF constructs was a generous gift from Dr. Jafari. Nitrocellulose membrane, peroxidase, mouse anti rhGM-CSF monoclonal antibody, LEGEND MAX™ human GM-CSF ELISA kit and 3, 3’-diaminobenzidine (DAB) were purchased from Wathman, UK, Abcam, UK, mybiosource, Biolegend, USA, Roche and Germany, respectively. 

*Cell culture*

CHO-K1 cells, as an adherent cells, were cultivated in 75 cm^2^ T flasks in 1:1 Dulbecco’s Modified Eagle Medium/Ham’s F12 (DMEM/Ham’s F12) supplemented with 10 % (v/v) fetal Bovine serum (FBS) and 1% (v/v) Penicillin/Streptomycin. Cells were incubated in an incubator with 5% CO2 at 37 °C. In order to adaptation, cells were detached and subcultured according to standard protocol. Briefly, culture medium was discarded and the cell layer was rinse with 0.25% (w/v) Trypsin- 0.53 mM EDTA solution to remove all traces of serum which contains trypsin inhibitor. Then 2 to 3 mL of Trypsin-EDTA solution was added to flask and cells observed under an inverted microscope until cell layer was dispersed. 6.0 to 8.0 mL of complete growth medium was added and aspirated cells by gently pipetting. Appropriate aliquots of the cell suspension was transformed to new culture vessels and incubated at 37 °C (14). 

*Transient transfection and stable cell transfectants generation*

Recombinant CHO cells were established by transfection of a vector (pcDNA3.1/Hygro+) containing the human Granulocyte-Macrophage Colony Stimulating Factor (GM-CSF) gene into CHO cells. Approximately 24 h before transfection, an appropriate number of CHO-K1 (3 × 10^5^) cells were seeded in 1 mL complete growth medium per well in a 6-well plate. Then, 1.0 μg plasmid DNA was added to 100 μL of serum-free medium and Mix completely by gently pipetting. 3.0 μL Lipofectamine Transfection Reagent was added to the diluted DNA mixture and gently mixed and briefly centrifuged to collect reaction mixture in bottom of the tube. Finally, the transfection complex drop-wise was added to the 6-well plates containing cells in complete growth medium and the cells were incubated overnight. For generating stable cell transfectants, cells were Passaged 48 h post-transfection in complete growth medium containing appropriate selection antibiotics (200 μg/mL Hygromycin) (14). 

*CHO adaptation from Serum-supplemented Media to Serum-free media*

Cells were cultivated in 10% (v/v) FBS supplemented DMEM-hams F12 to a density of 2 × 10^6^ cells/mL and were subcultured to a density of 2 × 10^5^ cells/mL into a 50:50 ratio of serum-free medium and the original serum supplemented medium. Cells were incubated at 37 °C and 5% CO2 until the viable cell density exceeds 2 × 10^6^ cells/mL and viability is greater than 90%. And were subcultured to a density of 2 × 10^5^ cells/mL into a 75:25 ratio of serum-free medium and the original serum supplemented medium. When the viable cell density exceeds 2 × 10^6^ cells/mL, with greater than 90% viability, were subcultured to 2 × 10^5^ cells/mL into 100% serum-free medium. DMEM-hams F12 medium without FBS was used as serum free part in two first adaptation steps and DMEM-hams F12 plus insulin, transferrin, selenium and glucose supplements according to Box–Behnken experimental design matrix was used as 100% serum free medium. 

*Protein expression analysis by gel electrophoresis*

 The supernatant of recombinant CHO culture mediums were harvested and concentrated with PEG 20,000 and dialyzed. Concentrated samples were dissolved in sample buffer contain SDS as denaturant and bromphenol blue as loading dye. 4% stacking and 15% separating acrylamide gel were prepared. Twenty microliter of each samples were loaded on the gel, along with molecular weight markers and power supply was set to 100 V. After 2 h, process was stopped and finally separated protein bands were stained and visualized with coomassie blue G250 and background of gel destained with glacial acetic acid and ethanol (15). 

*Western blot analysis of rhGM-CSF*

The expression of glycosylated rhGMCSF was confirmed by western blotting. The proteins resolved by SDS-PAGE were transferred to a nitrocellulose membrane and the membrane was blocked using Phosphate buffered saline with 5% nonfat milk. The blocked membrane was then immersed in 1:2000 dilution of anti rhGM-CSF monoclonal antibody conjugated to peroxidase for 2 h at room temperature. The immunoreactivity was visualized using chromogenic substrate 3, 3’-diaminobenzidine (DAB) (5).

*Protein concentration assay*

**Figure 1:**
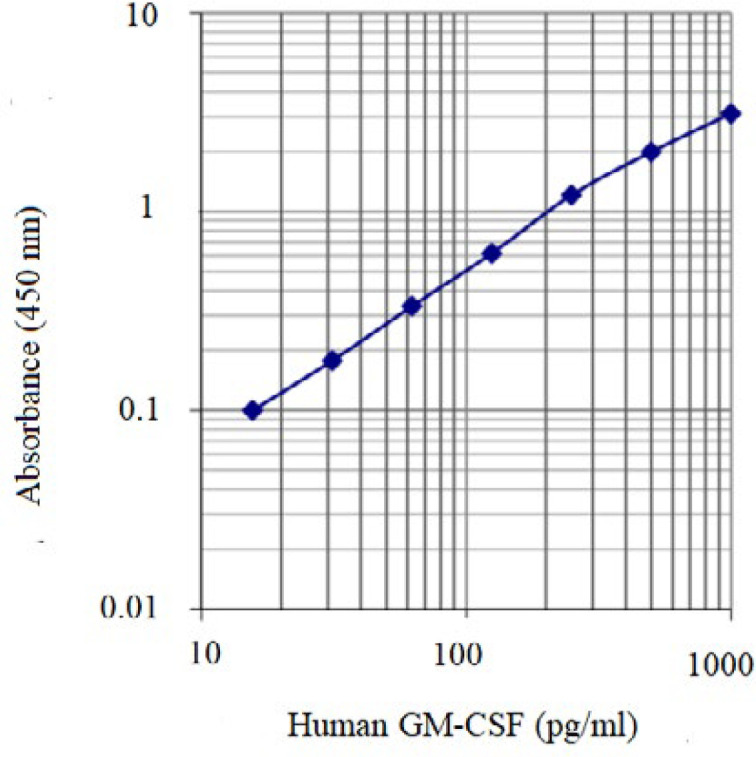
Standard curve for concentration measurement of human GM-CSF by ELISA protocol

The Concentration of the rhGM-CSF in samples harvested from cell culture supernatant was measured by LEGEND MAX™ human GM-CSF ELISA kit according to the manufacturer’s protocol provided. In brief, the anti rhGM-CSF antibody was bounded onto a micro-well plate and thereafter it was interacted with rhGM-CSF. The rhGM-CSF was connected to a secondary antibody that was in turn linked via conjugation to Horseradish peroxidase (HRP) enzyme. The detection of the assay was carried out by evaluating the HRP activity of the conjugate which was implemented by incubation with a suitable substrate for the enzyme resulting in a generation of a colored product (yellow product). Absorbance was read at 450 nm within 30 min. Finally, according to sample optical density (OD) and by standard curve, rhGM-CSF concentration was calculated. According to manufacturer’s protocol, for standard curve drawing 15.6 pg/mL, 31.3 pg/mL, 62.5 pg/mL, 125 pg/mL, 250 pg/mL, 500 pg/mL and 1000 pg/mL concentrations of human GM-CSF standard were prepared. Standard samples were processed based on above described protocol and OD of standards was read and standard curve was obtained based on OD and GM-CSF concentration (Figure 1). 

*Box-Behnken Experimental design*

Design-Expert software version 11, Stat-Ease Inc. was used to design current experiment. Box–Behnken experiment design as response surface methodology was used to study the effects of the four variables on the recombinant rhGM-CSF production. The variables were initial concentrations of insulin (A), transferrin (B), selenium (C) and glucose (D). The low, center and high levels of each variable are designated as -1, 0, and 1, respectively as illustrated in Table 1. The experimental levels for each variable were selected based on literature review and the results from our pilot studies. The response function was recombinant rhGM-CSF concentration. In Box–Behnken methodology the total number of experiments (N) is N = 2k (k-1) + r, where (k) is the number of variables and (r) is the replicate number of the central point. A total of 27 experiments include three replicate of the central point have been employed in this study to evaluate the effects of the four key factors on rhGM-CSF production by CHO Cells in designed serum free culture medium. The actual experimental design matrix is given in Table 2.

The performance of the process was evaluated by analyzing the response (y), which depends on the input factors A, B, C and D and the relationship between the response and the input process parameters. For RSM, the most commonly used second-order polynomial Equation developed to fit the experimental data and determine the relevant model terms can be written as: 

y = β_0 _+ ∑ β_i_X_i_+∑ β_ii_X^2^_i _+ ∑ β_ij_X_i_X_j_. Where β_0 _is the constant coefficient, β_i_ is the linear effect of the input factor X_i_, β_ij_ is the linear by linear interaction effect between the input factors X_i_ and, β_ii_ is the quadratic effect of input factor X_i_.

## Results

*Detection and identification of rhGM-CSF*

**Figure 2:**
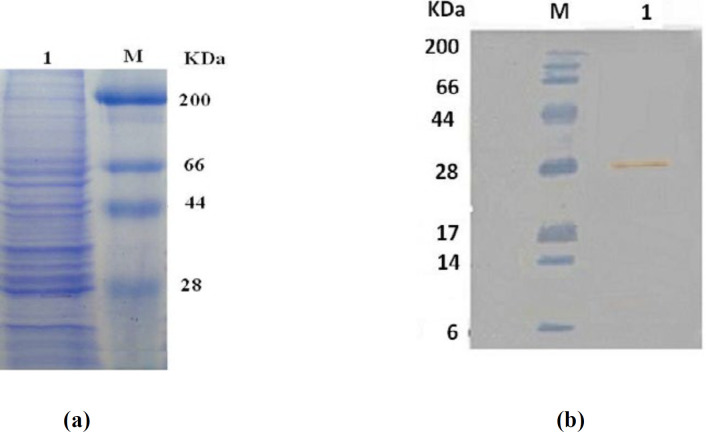
(a) rhGM-CSF protein expression analysis by SDS-PAGE Lane M, molecular weight marker (kDa) Lane 1, rhGM-CSF protein, (b) rhGM-CSF protein expression was confirmed by Western blot analysis Lane M, molecular weight marker (kDa) Lane 1, rhGM-CSF protein

Recombinant CHO Cells were grown in designed serum free medium containing different amounts of key factors based on the Box-Behnken experiments and 1% penicillin/streptomycin. To confirm the expression and identity of produced rhGM-CSF, supernatants were collected and expression of rhGM-CSF and secretion of it in the medium were showed as a 28 kDa band in SDS-PAGE gel electrophoresis (Figure 2a) and then were confirmed by Western blot analysis. Analysis of the rhGM-CSF by Western blot and detection with a monoclonal antibody against rhGM-CSF showed a 28 kDa band corresponding to rhGM-CSF (Figure 2b).

*Box-Behnken statistical analysis*

The Box–Behnken responses were analyzed and the ANOVA results for rhGM-CSF production in serum free designed medium are presented in Table 3. The analysis of variance is essential to test significance and adequacy of the model. The Model F-value of 44.79 is greater than the F-value obtained from the standard distribution table, confirming the adequacy of the model fits. *P*-values less than 0.05 show model terms are significant. In this case A, B, C, D, A², B², C², D² are significant model terms. The Lack of Fit *P*-value > 0.05 shows the Lack of Fit is not significant. The non-significance lack-of-fit showed that the model was valid for this experiment.

**Figure 3:**
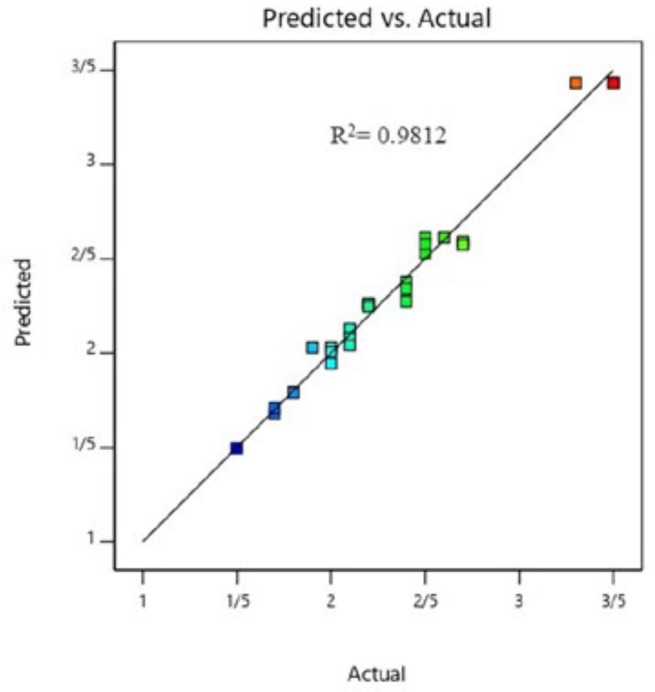
Plot of the experimental and predicted responses. High R^2^ value of the model indicated a good fit between model predicted values and the experimental data

The coefficient of determination (R^2^) of the model was 0.9812, which indicated a good fit between predicted values and the experimental data points (Figure 3). In addition, this shows that 98.12% of the variations for rhGM-CSF production are describe by the independent variables, and this also means that the model does not explain only about 1.88% of variation. Predicted R^2^ is a measure of how good the model predicts a response value. In current work the Predicted R² of 0.9054 is in reasonable agreement with the Adjusted R² of 0.9593, it means the difference is less than 0.20. If they are not, there may be a problem with either the data or the model.

In this work the models have high R^2^ value, significant F-value, an insignificant lack-of-fit *P*-value and low standard deviation (SD = 0.10) and coefficient of variance (CV = 4.5). These results indicate the high precision in predicting the rhGM-CSF production efficiency by selected four key factors. Therefore, the models were used for further analysis.

*Effect of interactive variables*

**Figure 4:**
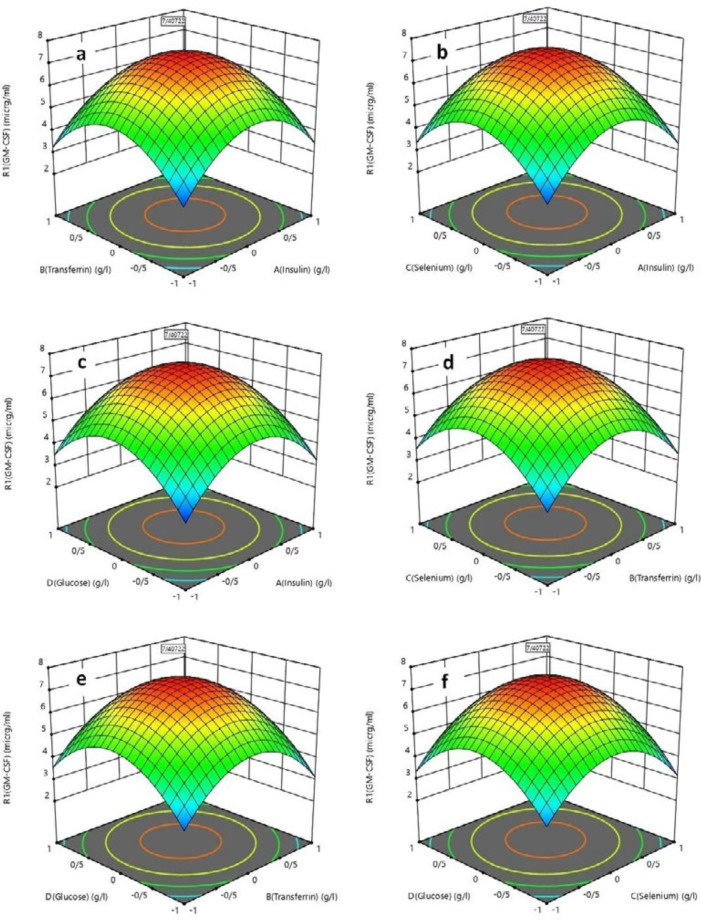
Response surface plots for rhGM-CSF production (g/L) in serum free medium: (a) effect insulin/transferrin (selenium 0.0007 g/L, glucose 1 g/L); (b) effect insulin/selenium (transferrin 0.5 g/L, glucose 1 g/L); (c) effect insulin/glucose (transferrin 0.5 g/L, selenium 0.0007 g/L); (d) effect transferrin/selenium (insulin 1 g/L, glucose 1 g/L); (e) effect transferrin/glucose (insulin 1 g/L, selenium 0.0007 g/L); and (f) effect selenium/glucose (insulin 1 g/L, transferrin 0.5 g/L)

The response surface plots of the second-order polynomial equation with two variables are given in Figure 4.

Figures 4a-4c present response surface plots of the effect of insulin and transferrin concentration, insulin and selenium concen-tration, and insulin and glucose concentration on the rhGM-CSF production respectively. It shows that rhGM-CSF concentration increased with increasing insulin concentration at 0-1 g/L but further increase in insulin concentration shows a decrease. Figure 4f explains the contour response surface plots showing the effect of selenium and glucose concentration on rhGM-CSF concentration. The rhGM-CSF concentration was increased with an increase in selenium concentration at 0–0.0007 g/L but further increase in selenium concentration shows a decrease. rhGM-CSF concentration increases along with an increase in glucose concentration from 0 to 1 g/L. Also, the increase in rhGM-CSF concentration with increasing amount of transferrin is shown in Figures 4d and 4e at 0-0.5 g/L.

*Optimization using the desirability function*

**Figure 5:**
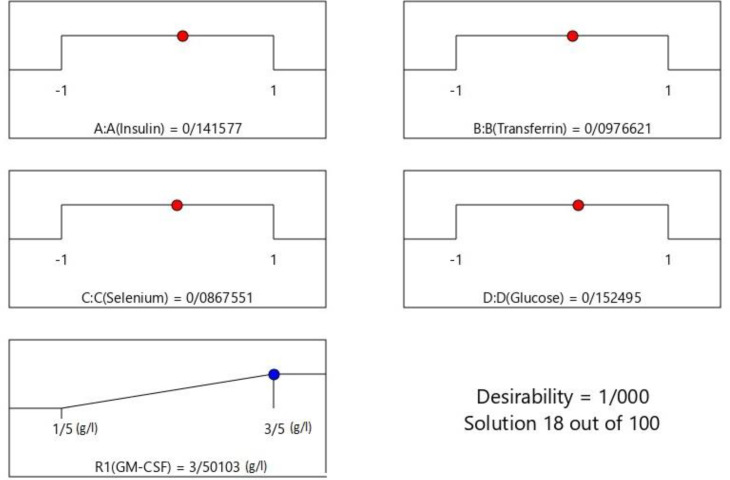
Desirability ramp for optimization. Maximum concentration of rhGM-CSF happened when the value of each variable is close to the central value

By using numerical optimization, a desirable value for each input factor and response can be selected. In, the possible input optimizations that can be selected include: minimum, maximum, in range, target, equal to (for responses) and set so as to establish an optimized output value for a given set of conditions. In this study, the input variables were given specific ranged values, whereas the response was designed to achieve a maximum. Using these conditions, the maximum achieved rhGM-CSF concentration was 3.5 g/L (Figure 5) at an insulin concentration of 1.1 g/L, transferrin concentration of 0.545 g/L, selenium concentration of 0.000724 g/L, and glucose concentration of 1.4 g/L. The confirmatory experiment showed a rhGM-CSF concentration of 3.5 g/L under optimal conditions compared with the rhGM-CSF concentration of 3.501 g/L obtained by the model. This indicates the suitability and accuracy of the model.

Finally, cultivation of recombinant CHO cells in a commercial serum free medium (CHOCLON, sinaclon company) was showed lower expression of rhGM-CSF (3.1 g/L) compared to our optimized serum free medium. 

## Discussion

The culture of CHO cells for recombinant proteins production in an industrial scale requires suitable media that support growth and production. Common media support high cell density and subsequent high expression of recombinant products but are Complex. Animal sourced components in these media affect downstream processes like purification and product quality (11). In present study we deigned a serum free medium with optimization of four effective factors insulin, transferrin, selenium and glucose to reduce complexity and undesired effects of serum component on product quality. According to the last studies glucose is an important source of energy for cell growth and protein production and insulin is essential for glucose transport into cultured cells. Optimum concentration of these factors is different in various reports. For example, we showed in 1.1 g/L insulin concentration we have maximum concentration of rhGM-CSF while Ce Gu *et al.* showed 1.0 mg/L insulin significantly increased human‐induced hepatocyte‐like cells proliferation and viability in Serum free medium (13). Gerhard Gstraunthaler showed selenium and transferin are necessary for cell growth (12). For example, selenium is important for activity of essential enzyme like glutathione reductase and glutathione peroxidase, catalyzing glutathione metabolism and turnover. Transferrin is a serum protein that is responsible for cellular iron transport (12). In this study Box-Behnken experimental design was used for optimization of these critical factors. rhGM-CSF concentration was a criteria for evaluation of results. The traditional approach to optimization of a process is to change 1 variable at a time. In this method it is difficult to optimize several variables with several levels, as the method reveals nothing about the interactions among the variables (16). Hence, a Box-Behnken statistical design with 4 variables, 3 levels, and 27 runs was selected for the optimization study. The experimental design consists of a set of points lying at the midpoint of each edge and the replicated center point of the multidimensional cube. In current study achieved maximum rhGM-CSF concentration was 3.5 g/L at an insulin concentration of 1.1 g/L, transferrin concentration of 0.545 g/L, selenium concentration of 0.000724 g/L, and glucose concentration of 1.4 g/L. In another study Hideo Miki *et al.* showed EX-CELL™302 serum free commercial medium, which contained an IGF-1 analog, supplemented with lysophosphatidic acid (LPA) resulted in increasing CHO growth rate and antibody production. Also, when IGF-1 was replaced with aurintricarboxylic acid (ATA) serum-free medium enhanced the growth of CHO cells and antibody production comparable to serum-containing medium in CHO suspension culture (17). Jian Xu *et al.* designed serum free medium for CHO cultivation. They studied seven factors including ZnSO_4_, transferrin, putrescine, bovine serum albumin (BSA), ferric citrate, sodium pyruvate, and ethanol amine using Plackett-Burman design and support vector machine (SVM) methodology and showed ZnSO_4_, transferrin, and bovine serum albumin (BSA) are more important supplements for CHO growth in serum free medium (18). Do Yun Kim *et al.* designed a serum free medium based on Iscove’s modified Dulbecco’s medium (IMDM) for antibody production in CHO cell line. In this study IGF-1 and ferric citrate were supplemented instead of insulin and transferrin. They showed cell density and specific antibody productivity increased from 7.6 × 10^6^ cells and 19.14 μg to 7.79 × 10^6^ cells d and 54.04 μg, respectively (19). 

**Table 1:** Independent variables and their levels used for Box–Behnken design

**Variables/Unit**	**Factors**		**Levels**	
		Low (-1)	Center (0)	High (1)
Insulin concentration (g/L)	A	0	1	2
Transferrin concentration (g/L)	B	0	0.5	1
Selenium concentration (g/L)	C	0	0.0007	0.001
Glucose concentration (g/L)	D	0	1	5

**Table 2:** Box–Behnken experimental design matrix and experimental responses. Box–Behnken methodology represent 27 experiments based on different concentration (g/L) of four variables

**Run**	**A(insulin) (g/L)**	**B(transferrin) (g/L)**	**C(selenium) (g/L)**	**D(glucose) (g/L)**	**R1(rhGM-CSF) (g/L)**
1	0	1	0	-1	2.1
2	0	0	0	0	3.5
3	0	1	-1	0	2.2
4	-1	0	0	1	2.1
5	0	-1	0	1	2.4
6	1	0	1	0	2.7
7	0	-1	-1	0	2
8	1	0	0	-1	1.9
9	0	0	-1	1	2.2
10	0	0	0	0	3.3
11	0	0	0	0	3.5
12	0	-1	1	0	2.2
13	1	1	0	0	2.6
14	1	0	0	1	2.5
15	-1	-1	0	0	1.8
16	0	1	1	0	2.5
17	0	0	-1	-1	1.7
18	0	0	1	1	2.5
19	1	-1	0	0	2.4
20	1	0	-1	0	2.4
21	-1	1	0	0	2.1
22	0	1	0	1	2.7
23	0	-1	0	-1	1.7
24	-1	0	-1	0	1.8
25	0	0	1	-1	2
26	-1	0	0	-1	1.5
27	-1	0	1	0	2

**Table 3:** Analysis of variance (ANOVA) for Response Surface Quadratic Model

**Source**	**Sum of Squares**	**df**	**Mean Square**	**F-value**	***P*** **-value**	
Model	6.75	14	0.4821	44.79	< 0.0001	
A-A(insulin)	0.8533	1	0.8533	79.28	< 0.0001	
B-B(transferrin)	0.2408	1	0.2408	22.37	0.0005	
C-C(selenium)	0.2133	1	0.2133	19.82	0.0008	
D-D(glucose)	1.02	1	1.02	94.84	< 0.0001	
AB	0.0025	1	0.0025	0.2323	0.6385	
AC	0.0025	1	0.0025	0.2323	0.6385	
AD	0.0000	1	0.0000	0.0000	1.0000	
BC	0.0025	1	0.0025	0.2323	0.6385	
BD	0.0025	1	0.0025	0.2323	0.6385	
CD	0.0000	1	0.0000	0.0000	1.0000	
A²	2.31	1	2.31	214.74	< 0.0001	
B²	1.59	1	1.59	147.62	< 0.0001	
C²	1.97	1	1.97	183.36	< 0.0001	
D²	2.77	1	2.77	257.45	< 0.0001	
Residual	0.1292	12	0.0108			
Lack of Fit	0.1025	10	0.0103	0.7687	0.6853	
Pure Error	0.0267	2	0.0133			
Cor Total	6.88	26				

## Conclusion

In this study, a serum free medium was designed for rhGM-CSF production by optimization of four key factors insulin, transferrin, selenium and glucose. These factors were selected by study and analysis of serum component and its effect on cell growth. The statistical methodology, Box–Behnken Response Surface design is demonstrated to be effective and reliable for optimization of factors involved in production of rhGM-CSF in serum free medium. The response surface plots were used for estimating the effect of four independent variables (insulin concentration, transferrin concentration, selenium concentration and glucose concentration) on the response (rhGM-CSF concentration). The second order mathematical model was developed by ANOVA analysis of the experimental data obtained from 27 experiment runs. 

Applying the method of the desirability function, optimization of insulin concentration of 1.1 g/L, transferrin concentration of 0.545 g/L, selenium concentration of 0.000724 g/L, and glucose concentration of 1.4 g/L gave a maximum of 3.5 g/L rhGM-CSF production with desirability of 1.0. This study demonstrated that CHO can grow well in serum free culture medium with optimized important factors and produce rhGM-CSF in an acceptable concentration. 
